# Mind Cure

**Published:** 1888-06

**Authors:** S. P. Crawford

**Affiliations:** Stockton, California


### MIND CURE.

By S. P. Crawford, A. M., M. D., Stockton, Cal.

By the heading of this paper, I mean those peculiar fanatical *isms* that profess to cure diseases through the influence of the mind alone, ignoring all other means. Spiritual, faith and prayer cures come under the same heading, for whatever results, good or bad, come from any of these methods, they are through the mind.

The medical profession is the guardian of health and is ever on the alert for the best methods of the prevention and cure of disease. Medicine is bounded by no circumscribed philosophy, *pathy* or *ism*. Her agencies are limited to no kingdom, material or immaterial.

Her *materia medica* are drawn from all sources, tangible and intangible. Special philosophies, *pathies* and *isms* are mere side shows in medicine, containing some truth, but infinitely less than the whole truth. I mean by these side shows, those various systems and so-called schools of medicine that have from time to time sloughed off from regular medicine and pitched their tents outside the camp. They usually have their origin in some cranky notion, or in prejudiced minds with narrow and contracted views, unwilling or incapable of grasping the whole truth. True medicine is bounded by no contracted philosophy, but is the sum of all that is true of all philosophies. To be less than this is to be less than a physician.

An ism or pathy having sloughed off of true medicine, carrying with it some truth, does not debar the true physician from using that truth as his own. Error we discard; but truth is ours by inheritance, wherever found. If it becomes necessary to treat certain diseases by the expectant plan with infinitesimal remedies, (which have no virtue only so far as they affect the mind) they are ours to use. If mineral, vegitable, or animal substances seem to be indicated, they are ours to use. If electricity, magnetism, mind force, or any other agent, tangible or intangible, can be of use, they are ours to use. No "pent up utica" limits investigation, no prejudice limits him to any agency, or set of agencies, in his labors as a physician.

The environments, pathological, physiological and psychological bearings must be taken in every case of disease. The physician is a *physician*, nothing more and nothing less. He knows no *pathy* or *ism*. To be any of these is to be less than a physician, for they limit his labors and duties to prescribed theories, prejudice, judgment and reason, and sink him to an infinitesimal speck on the medical horizon. Every disease in every case is a problem of itself to be studied and worked out, not according to set formulas or preconceived notions, but by a thorugh investigation and knowledge of all bearings in that particular case. Having satisfied himself of all the conditions of that special case in that particular organism, he selects the best and most reliable agents from the vast domain of agencies, that in his experience or that of others are best adapted to that disease and that particular organism.

To simply diagnose a disease, and then indiscriminately use remedies said to be good in such disease, without regard to environments, conditions, peculiarities and idiosyncrasies, is shooting in the dark. To be ab e to diagnose a disease and have but one remedy for that disease in all cases and conditions, is criminal folly. And above all, to have but one remedy for all diseases and all conditions is nothing more nor less than to be a murderous crank.

In the last category comes that class of fanatics who profess to cure all diseases through the mind alone. This brings me to the heading of this article.

Mind cure, faith cure, and prayer cure, all come under the same physiological explanation. It is claimed that, in the latter, cures are effected by Divine agency, while in the two former, the mind alone does the work. But it will be seen that t e *modus operandi* is the same in all cases. There are mind and prayer cure establishments in various parts of the country, and every case of recovery that occurs in these establishments is usually heralded from one end of the land to the other.

It would be interesting to know how many failures there are, and how many deaths take place to one recovery. The failures and mortuary statistics of such places we never hear of. These mixed cure establishments I look upon as so many "dead falls" of cranks. Where one recovers from an imaginary disease or functional disorder, fifty no doubt fail to be benefited, or die for the want of rational treatment.

Whatever virtues there may be in the mind, it is incompetent to reach surgical cases, organic or germinal diseases, and never of itself cures anything, except, perhaps, some purely imaginary and functional disorders. That the mind has an influence over the body in health and disease no one will question, and I regret that *mental therapeutics* is so often ignored and poo'd at by the physician. I set forth mind force as an important factor in the hands of the physician in treating diseases, in a paper on *Psychotherapeutics* read before the *San Joaquin County Medical Society* some years ago, and subsequently published in the *Nashville Journal of Medicine and Surgery.* I shall draw upon that paper for much of the subsequent material of this paper. The mind is an invaluable help to the physician in the sick room, and should be wrested from the hand of charlatans. In all cases of adults, where cure is possible, it is an invaluable aid to remedial agents. In cases of nervous and functional disorders, the mind alone has been known to cure. It is in such cases as these, which, to the uninformed, present grave symptoms, that charlatans and fanatics perform their wonderful cures, which to the credulous and superstitious, seem nothing short of miracles. Belief or faith inspires hope, and hope is tonic and stimulant and a valuable adjuvant in the hands of the physician· The physician's first aim should be to inspire confidence, then he has complete control of the mental forces.

> Hope "Thine is thec harm of lips bewildered way.
> That calls each slumbering passion into play."

Hope arouses the flagging energies, equalizes the circulation, and in conjunction with material agents, aids the eliminating powers of the system, and recovery is more certain. Fear, which is the reverse or negative condition of the mind, has been known to kill, by the sudden depression or suppression of nervous energy. These intangible forces are no mere phantoms of the imagination, but *substantive* realities that cannot be ignored in our dealings with the sick. Remedial agents may properly be divided into two classes, *objective* and *subjective*, the former acting from without, the latter from within. It is important for the physician to know just when and how to handle these subtle subjective forces, as it is to know when and how to handle objective or material agents. Hope and confidence are transitory in their effects, and must be sustained by objective forces, hence material agents are demanded in nearly all cases of disease. save, perhaps, in those of a purely imaginary or functional character, already alluded to, in which the mind alone has been known to effect instantaneous cures. Here lies the true solution of those *miraculous* cures claimed to be performed by mind curers, or through Divine agency. There is neither mystery nor supernatural agency about them, as any physician well knows.

Since the "ancient of days," mind has been recognized as a potent factor in the healing art. "He doth more cures in whom most trust," was an aphorism of Hippocrates, which clearly demonstrates that the father of medicine recognized mind as an important factor in the cure of disease. Every physician of any experience, has often witnessed the powers of the mind inspired by confidence, especially in the lying in room, and knows full well the advantages of assurance in such cases.

How often has the physician seen, on entering the lying-in room, his patient in the most intense pain and anxiety from the dread of "something wrong." So intense was this fear and anxiety, that the labor pains were erratic and futile, only intensifying her agony· Upon an examination and the cheerful assurance that all was right' and there was no trouble or danger, hope seized the helm, mental agony ceased as with the magician's wand, and labor assumed its regular and legitimate work. The presence, often, of the trusted physician is sufficient to restore nature to its normal condition. To better illustrate my meaning, I will cite a single case. I was called by the attending physician to assist him in what he supposed would have to be instrumental labor. The case was one in which the physician had failed to inspire confidence, and in fact, was in almost as great a state of perturbation of mind as his patient. Upon entering the room I found the patient in great anxiety and distress, which my presence only intensified, for the necessity of a consulting physician only confirmed her worst fears. Her pains had ceased, or were not felt in her mental agony. Upon an examination, I found the os completely dilated, and nothing whatever to indicate the use of instruments. I gave her postive assurance that there was no danger and that labor would soon terminate favorably, got in my buggy and drove home. In less than thirty minutes she fell into a gentle slumber which lasted an hour, from which she awoke, had a few pains and all was over. The patient told me afterwards, that in all her experience, (having borne several children before,) she had never suffered such anxiety of mind and pain of body, and that my assurance did little to releive her at the time, but when she found I had gone away, it confirmed her in my assurance, for surely, she reasoned, "he would not go home if I was in danger." "I felt a calmness come over me at this thought," she said, "and fell asleep, from which the pains awoke me, without fear or anxiety." In this case there was no abnormal condition of body, save that brought about by the abnormal condition of mind, which when restored, nature was herse.f again.

The mind may be manipulated by various methods, by positive assurances, by the exercise of faith in prayer, by any method that will inspire confidence, hope and trust. The *modus operandi* of these mental conditions are the same, no difference from what method they are produced. Prayer, with the religiously disposed, is an invaluable method of producing equinam ty of mind, begetting hope and trust. I shall not attempt any theological discussion of the Divine agency in prayer, but this much I will say, as viewed from a medical stand point, that prayer is not *objective* affecting the Divine will, by which it is moved to send some supernatural force in answer to it. Prayer, in the writer's judgment, is *subjective*, acting upon the mind and system through the inspiration of hope and trust. It conveys to Deity no knowledge that he did not know, creates no new emotion in the breast of the Sovereign, does not alter His will or laws, but simply bring the mind into closer relations and obedience to His laws and will, from which all blessings come through the natural order of things. God couples his blessings with man's agency. Prayer for the comforts and blessings of life or a bountiful harvest, will add nothing to the store house of him who does not work, plough nor sow. Prayer for the blessings of health, or the removal of disease, will fail where the means at hand are not used. To pray and then fold the arms in expectation of some miraculous power to follow, is in effect saying to the Almighty, now I have done my duty, do yours.

These prayer cure establishments are the hot beds of superstition, degrading to religion, fanatical in idea—*misconceptions* to say the least of them, of man's duties and relations to Deity. A trust in God, with an intelligent use of the means at our command, are the assurances of success in all honest departments of business, professions and avocations in life.

